# Home-based self-management multimodal cancer interventions & cardiotoxicity: a scoping review

**DOI:** 10.1186/s40959-024-00204-6

**Published:** 2024-02-29

**Authors:** Anna Talty, Roseanne Morris, Carolyn Deighan

**Affiliations:** The Heart Manual Department, Astley Ainslie Hospital, Grange Loan, Edinburgh, Scotland, UK EH9 2HL

**Keywords:** Cardiotoxicity, Cardio-Oncology, Cancer Interventions, Self-Management, Home-Based, Multimodal

## Abstract

**Background:**

Due to advancements in methods of cancer treatment, the population of people living with and beyond cancer is dramatically growing. The number of cancer survivors developing cardiovascular diseases and heart failure is also rising, due in part to the cardiotoxic nature of many cancer treatments. Guidelines are being increasingly released, emphasising the need for interdisciplinary action to address this gap in survivorship care. However, the extent to which interventions exist, incorporating the recommendations of cardio-oncology research, remains undetermined.

**Objective:**

The aim of this scoping review is to assess the nature, extent and remit of existing cancer care interventions and their integration of cardio-oncology principles.

**Methods:**

The review was conducted in accordance with the PRISMA Extension for Scoping Reviews Guidelines. Databases were independently searched for articles from 2010 to 2022, by two members of the research team. Data were charted and synthesised using the following criteria: (a) the focus of the intervention (b) the medium of delivery (c) the duration (d) the modalities included in the interventions (e) the research articles associated with each intervention (f) the type of studies conducted (g) key measures used (h) outcomes reported.

**Results:**

Interventions encompassed six key modalities: Psychological Support, Physical Activity, Nutrition, Patient Education, Lifestyle and Caregiver Support. The focus, medium of delivery and duration of interventions varied significantly. While a considerable number of study protocols and pilot studies exist documenting HSMIs, only 25% appear to have progressed beyond this stage of development. Of those that have, the present review did not identify any ‘feasible’ interventions that covered each of the six modalities, while being generalisable to all cancer survivors and incorporating the recommendations from cardio-oncology research.

**Conclusion:**

Despite the substantial volume of research and evidence from the field of cardio-oncology, the findings of this scoping review suggest that the recommendations from guidelines have yet to be successfully translated from theory to practice. There is an opportunity, if not necessity, for cardiac rehabilitation to expand to meet the needs of those living with and beyond cancer.

## Background

Due to advancements in methods of cancer diagnosis and treatment, the population of people living with and beyond cancer is dramatically growing. In the UK, cancer survival has doubled in the past 40 years [1]. In the US, projections estimate that the percentage of people living 5 or more years after their cancer diagnosis will increase by approximately 30% in the next decade [2]. The necessity for survivorship care is becoming increasingly urgent as a result, and further exacerbated by the prevalence of comorbidities within this demographic [3], due in part to the harmful effects of certain anticancer treatments [4]. Many cytotoxic treatments are associated with ‘cardiotoxic’ effects, incurring damage to the cardiovascular system [5] which can accelerate the development of cardiovascular diseases and heart failure [6] analysis by Stoltzfus et al. [7] found that fatal heart disease is over twice as likely to occur in cancer patients, compared to the general population.

It is therefore unsurprising that the field of cardio-oncology, which refers to the management of the cardiovascular health of cancer patients, has been insisting on the exigency of developing evidence-based methods of preventing, detecting, monitoring and treating cardiac risk factors in this vulnerable population. Guidelines and scientific statements have been released by the European Society of Cardiology [8], European Society for Medical Oncology [9], International Cardio-Oncology Society [10], American Heart Association [11] and the Canadian Cardiovascular Society [12] emphasising the need for interdisciplinary action to address this gap in survivorship care.

One discipline that is well-positioned to potentially influence and guide the development of interventional cancer care, is cardiac rehabilitation. Emerging evidence supports the efficacy of behavioural interventions for patients exposed to cardiotoxicity [13] and within cardiac rehabilitation, home-based self-management programmes facilitating lasting health behaviour changes for cardiac patients are well-evidenced and well-established. These interventions are evidence-based and multimodal in nature, meaning they encompass several factors necessary for a comprehensive recovery, such as physical, nutritional, psychological and social components. A substantial body of literature documents the efficacy of these programmes [14] and certain interventions (e.g., The Heart Manual [15, 16]) have received endorsement by NICE Guidelines [17].

However, it is as of yet unclear if such an equivalent exists for cancer patients – despite these programmes’ obvious relevance, particularly for cancer patients with underlying or cytotoxic-induced cardiovascular risk. A previous review indicated the lack of a comparable resource for cancer patients, concluding “No […] resource solely dedicated to treatment of chronic disease risk behaviours (smoking, obesity, physical inactivity, treatment nonadherence) exists in current models of integrated care” [18], p. 840] There has certainly been a surge in interest in home-based, self-management, multimodal interventions (HSMIs) for cancer patients in the past decade but these interventions appear to vary significantly in their definitions of key terms (e.g., what constitutes ‘multimodal’, target areas and populations, implementation success and evidence-base. The extent to which the interventions incorporate the recommendations of the cardio-oncology research and guidelines also remains undetermined.

### Objective

The aim of the present scoping review is to assess the nature, extent and remit of existing HSMIs, potential obstacles to their implementation, and to clarify key terms and concepts within the field. The findings of this review will be discussed in the context of the current literature on cardio-oncology and would be relevant to the progression of future developments in the field.

## Methods

The PRISMA-ScR Guidelines [19] were employed to inform the design of this scoping review’s methodology.

### Protocol and Registration

A preliminary literature search was conducted by a member of the research team in August 2022. On the basis of these findings, a study protocol for the present review was developed. The protocol was registered with the Open Science Framework on 13th December 2022 and can be accessed at: https://osf.io/gfcda.

### Eligibility criteria

In order to gain comprehensive insight into the scope of existing HSMIs for cancer patients, the following study types were included in the scoping review: randomised controlled trials, non-randomised controlled trials, grey literature, study protocols, pilot studies, exploratory trials, experimental studies, systematic reviews and meta-analyses of interventions, and the studies within. Articles focusing on pre-, during and post-treatment interventions, delivered through web-based (digital), phone-based and print (paper) mediums, and both self-guided and facilitator-led interventions were eligible for inclusion. Abstracts were also included, provided the intervention was described in sufficient detail to meet inclusion criteria.

Exclusion criteria consisted of interventions that were not home-based (i.e., based in a hospital or external centre), interventions that were not ‘multimodal’ in nature, meaning they only focused on one aspect of cancer care (e.g., diet or exercise or fatigue) and interventions that did not contain a self-management component. Only articles written in English were included. There were no restrictions applied regarding location or country of origin of the intervention. Articles published prior to 2010 were also excluded. Interventions targeting child and adolescent populations were excluded; only those aimed at adults (over the age of 18 years old) were included.

### Information sources and search

A search term was developed using Boolean Operators and refined by testing in multiple databases, before being reviewed by a senior member of the research team. The following was determined as the final search term: ‘(home-based OR "home based" OR telehealth OR remote OR web-based OR "web based") AND (self-managed OR self-management OR patient-led) AND (comprehensive OR multimodal OR multidimensional OR compound OR intermodal OR multilayered) AND (programme OR intervention OR prehabilitation OR rehabilitation OR survivorship OR "patient education") AND cancer’. The same search strategy was used for six databases searched: MEDLINE, EMBASE, PsychInfo, Cinahl, Cochrane Library, and AMED. The searches were conducted independently by two members of the research team. Each reviewer exported search results into a separate Microsoft Excel spreadsheet, before comparing the number of results generated from each database to ensure 100% consistency. The literature searches were conducted in January 2023, databases were searched from 2010 to 2022.

### Selection of sources of evidence

The reviewers independently removed duplicates and screened study titles and abstracts in order to ascertain articles eligible for full-text assessment. Where a systematic review or meta-analysis was deemed eligible, the articles within were extracted and also screened.

After completing the initial screening phase, the reviewers compared results to assess consistency. A number of discrepancies were observed at this stage. The reviewers discussed each case and where they were unable to reach a resolution by consensus, they brought the article to the senior member of the review team. A data coding form was then developed to standardise the full-text assessment process. The coding form was based on the eligibility criteria, it asked if the intervention was (a) home-based (b) included a self-management component (c) multimodal (d) for adults (e) published later than 2010. The same process of independent assessment followed by comparison and resolution of disagreements by the third party member was adhered to. In cases where data was missing, and it was not possible to verify if the intervention met the inclusion criteria, papers were excluded.

### Data charting process

The research team used the coding form, the eligibility criteria and research objectives to derive a data charting form in an iterative manner. It was decided that in order to answer the research questions posed by the present study, the chart should incorporate details of both the nature of the HSMIs and the nature of the research underpinning the interventions. The following data items were identified as being necessary to include: (a) the focus of the intervention (e.g., cancer type/symptom) (b) the medium of delivery (c) the duration (d) the modalities included in the interventions (physical activity, nutrition, etc.) (e) the research articles associated with each intervention (f) the type of studies conducted (g) key measures used (h) outcomes reported. The final data charting form was developed to consolidate this information, which the two reviewers independently used to extract the relevant information from the final selection of articles.

### Synthesis of Results

PRISMA-ScR Guidelines [19] were adhered to when creating the synthesis of results. The findings of the literature search and screening were presented using a PRISMA Flow Diagram and the key characteristics of the sources of evidence were displayed in tabular format, using the information extracted by the data-charting process. Narrative and visual representations of key findings were employed where appropriate. Due to the different terminology used in the literature to describe the same core modality (e.g., nutrition, diet, food, healthy eating), a thematic analysis was conducted to decipher the primary components incorporated by interventions. Given the remit of a scoping review (as outlined in the PRISMA-ScR Guidelines), it was not considered relevant to conduct a critical appraisal of the individual sources of evidence. Where studies reported key measures and outcomes, these were included in the data synthesis to provide an overview of the nature and effects observed by the available research. The included articles were searched for references to cardio-oncology principles and the final results were discussed in the context of the recent guidelines and literature on cardio-oncology and cardiotoxicity.

## Results

**Fig. 1:**
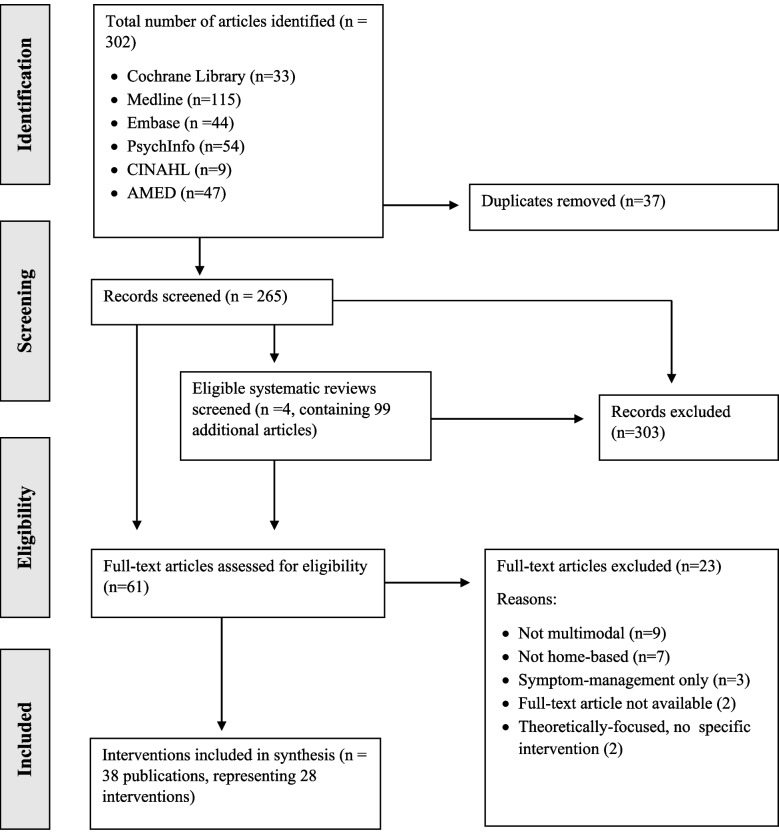
PRISMA flow diagram

The literature search retrieved 302 studies, 265 of which remained after the removal of duplicates. The titles and abstracts of the 265 studies were screened. Four eligible systematic reviews were identified and the 99 articles within screened. Following this, 61 studies underwent a full-text assessment. A further 23 articles were excluded, for reasons highlighted in Fig. 1. Thirty-eight publications, representing 28 distinct interventions were included in the final synthesis of results.

### Characteristics of interventions

The focus of each intervention is described in Table 2, together with the medium of delivery, duration, modalities included, research articles associated, type of studies conducted, key measures used and outcomes reported.

All interventions included were home-based, mediums of delivery varied from web-based (*n* = 13), telehealth conferencing (*n* = 9), telehealth conferencing with web- or mobile-based components (*n* = 4), mobile-based (*n* = 1) and paper-based (*n* = 1). The duration of interventions was diverse, ranging from four weeks (*n* = 1) to 24 weeks (*n* = 6). Fifteen interventions lasted between six and 16 weeks. Five interventions were missing data regarding their length. Only one intervention focused on cancer ‘prehabilitation’, its duration being 1–2 weeks prior to surgery [20]. The USA was the country of origin of the most interventions (*n* = 12), followed by Korea (*n* = 4), the Netherlands (*n* = 4) and Australia (*n *= 3). Canada and the UK were the location of two interventions, respectively. One intervention was based in Denmark [20].

The most common target areas of the interventions included were cancer-related fatigue (*n* = 5) and prostate cancer (*n* = 5), followed by any cancer type (*n* = 4), breast cancer (*n* = 4) and haematological cancers (*n* = 4). The remaining categories; abdominal cancer, bone marrow transplant, cancer with HIV, cancer-related cognitive impairment, cancer-related pain and head/neck, colorectal, breast, Hodgkin and non-Hodgkin lymphoma, were each the focus of one intervention.

### Intervention components

The components included by each of the 28 HSMIs were extracted, and an inductive thematic analysis was conducted in order to derive six key modalities encompassed by the interventions, the results of which are presented in Table 1. All interventions were multidimensional in nature and comprised of Psychological Support in conjunction with at least one other modality (Physical Activity, Nutrition, Patient Education, Lifestyle and Caregiver Support).

**Table 1:** Key components encompassed by interventions

Modalities	Components
Physical Activity	Exercise
	Physical Activity
Nutrition	Diet
	Healthy Eating
	Food
	Nutrition
Psychological Support	Mood
	Mindfulness
	Psychoeducation
	Distress Management
	Stress
	Emotional Concerns
	Cognitive Strategies (e.g., memory & attention)
	Depression
	Thoughts and Feelings
	Behaviour Change Techniques
	Relaxation
	Psychological Principles
	Telephone Counselling
	Cognitive Behavioural Therapy
	Mindfulness-based Cognitive Behavioural Therapy
	Self-Management Skills
	Peer Support
Patient Education	Energy Conservation
	Residual Symptoms
	Physical Side Effects
	Symptom Management
	Medication
	Constipation Management
	Topics in Tumour-Specific Modules
	Cardiovascular, Bone, Second Cancer Advice
	Treatment Type & Side Effects
	Pain Management
	Patient Information
	Urinary, Bowel, Sexual & Hormonal Problems
	Follow-Up Care
Lifestyle	Return to Work
	Relationships
	Smoking & Alcohol Cessation
	Sleep & Fatigue
	Communication
	Finance Management
	Home & Work Life
	Sexuality
Caregiver Support	Caregiver Guides
	Couples-Focused Interventions

**Fig. 2:**
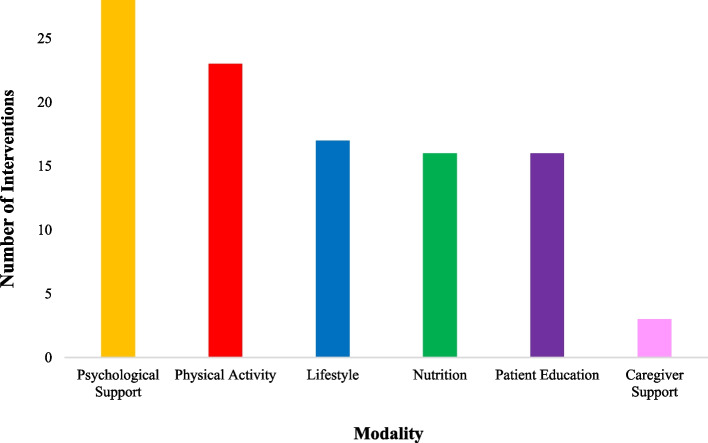
Frequency with which modalities featured in HSMIs

After ‘Psychological Support’, the most common modality across interventions was Physical Activity (*n* = 24), followed by Lifestyle (*n *= 17), Patient Education and Nutrition (*n* = 16, respectively), only three interventions included Caregiver Support, as seen in Fig. 2 below.

**Fig. 3:**
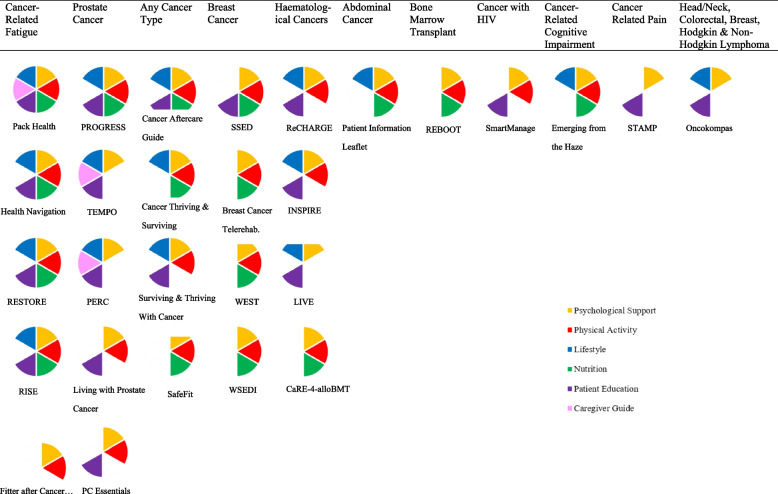
Modalities comprising HSMIs, according to intervention focus

Only one intervention ‘Pack Health’ [21] featured all six modalities. Five interventions featured five modalities (Caregiver Support was the modality not included in each). Ten interventions featured combinations of four modalities, 10 featured three, and only two featured two modalities: STAMP [22] which consisted of Psychological Support and Patient Education and ‘Fitter after Cancer’ & Less Tired from Cancer’ [23] which consisted of Psychological Support and Physical Activity. Figure 3 displays the modalities that comprise each intervention.

### Study characteristics & outcomes

Of the 38 studies included, 15 were study protocols, five were pilot or feasibility studies, nine were original research articles, one was a retrospective analysis and eight were randomised control trials (RCTs). The literature search only identified further research associated with four of the 16 study protocols [24–27], 3% of study protocols were not associated with later research. Similarly, no further research was identified in the literature search for the five pilot or feasibility studies.

Forty-six different outcome measures were employed by the included studies. These are listed in full in Table 2. Only 13 of the 28 interventions were supported by research investigating their efficacy. To summarise the preliminary evidence available, post-intervention, increases were observed in the following domains: emotional and social functioning, physical activity, fruit and vegetable intake, cognitive function, anxiety, global quality of life, health-related quality of life, fatigue self-efficacy and motivational readiness. Post-intervention, decreases were observed in the following domains: depression, fatigue*,* distress, symptoms, practical concerns, insomnia, body fat percentage and waist circumferences and positive coping. Smoking and patient activation were identified as two domains that did not demonstrate changes following participation in the interventions.

**Table 2:** Summary of studies included in the scoping review

*Summary of Intervention*					*Summary of Associated Research*			
**Title**	**Cancer Type/Aspect Targeted**	**Mode of Delivery**	**Length**	**Dimensions Targeted**	**Article Title**	**Study Type** *(Author, Location)*	**Outcome Measures**	**Results**
**Breast Cancer Telerehabilitation**	Breast Cancer	Telehealth	Missing data	Physical Activity, Nutrition, Patient Education, Peer Support, Emotional Support	Meeting the Rehabilitation and Support Needs of Patients With Breast Cancer During COVID-19: Opening New Frontiers in Models of Care	**Original Article** *(Binkley *et al*. *[28]*, USA)*	FACT-B, PSFS, UEFI	3 case studies were described to summarise the process of telerehabilitation. Results indicated telerehabilitation may be helpful in meeting breast cancer patents’ needs
**‘Cancer Aftercare Guide’** *(‘Kanker Nazorg Wijzer’)*	Any cancer type	Web-based	Missing data	Fatigue, Return to Work, Mood, Relationships, Physical Activity, Nutrition, Smoking, Residual Symptoms	The Kanker Nazorg Wijzer (Cancer Aftercare Guide) protocol: the systematic development of a web-based computer tailored intervention providing psychosocial and lifestyle support for cancer survivors	**Study Protocol** *(Willems *et al*. *[24]*, The Netherlands)*	N/A	N/A
					Lifestyle-related effects of the web-based Kanker Nazorg Wijzer (Cancer Aftercare Guide) intervention for cancer survivors: a randomized controlled trial	**RCT** *(Kanera *et al*. *[29]*, The Netherlands)*	CIS, EORTC QLQ-C30, HADS,	The intervention was effective in increasing emotional and social functioning and decreasing depression and fatigue 6 months after baseline
					Short-term effectiveness of a web-based tailored intervention for cancer survivors on quality of life, anxiety, depression, and fatigue: randomized controlled trial	**RCT** *(Willems *et al*. *[30]*, The Netherlands)*	DMIRSSC, DSQFC, SQUASH,	Moderate increases in physical activity and vegetable intake were demonstrated but did not remain significant after correction for multiple testing. No effect was found on smoking behaviour
**‘Cancer Thriving and Surviving’**	Any cancer type	Telehealth	6 weeks	Fatigue, Sleep, Psychoeducation, Nutrition, Relationships & Communication, Physical Activity, Weight, Depression	Enhancing Cancer care of rural dwellers through telehealth and engagement (ENCORE): protocol to evaluate effectiveness of a multi-level telehealth based intervention to improve rural cancer care delivery	**Study Protocol** *(Pal *et al*. *[31]*, USA)*	N/A	N/A
**‘CaRE-4-alloBMT’**	Haematological Cancer	Telehealth	6 months	Physical Activity, Nutrition, Psychosocial Distress, Self‐Management Skills	Cancer Rehab Program for Allogenic Bone and Marrow Transplant Patients—CaRE-4-alloBMT	**Study Protocol** *(Jones *et al*., *[32]*, Canada)*	N/A	N/A
**‘Emerging from the Haze’**	Any cancer type *(focus on CRCI)*	Telehealth	6 weeks	Psychoeducation, Stress Management, Physical Activity, Nutrition, Sleep, Cognitive Strategies (e.g., Memory, Attention)	Cancer-Related Cognitive Impairment: Retrospective analyses of a multidimensional, psychoeducation-based cognitive rehabilitation intervention	**Retrospective Analysis** *(Asher *et al*. *[33]*, USA)*	FACT-Cog, SF-36	Results demonstrated a significant improvement for self-reported cognitive function and HRQOL which was sustained over 12 months
					Emerging From the Haze: A Multicenter, Controlled Pilot Study of a Multidimensional, Psychoeducation-Based Cognitive Rehabilitation Intervention for Breast Cancer Survivors Delivered With Telehealth Conferencing	**Pilot Study** *(Myers *et al*. *[34]*, USA)*	FACTCog, MDASI, PROMIS-29, UCLA-LS,	The intervention was associated with participants’ improved perceived cognitive function up to 12-months post-intervention
**‘Fitter after Cancer’** and** ‘Less Tired from Cancer’** *(‘Fitter na Kanker’ and ‘Minder Moe Bij Kanker’)*	Any cancer type *(focus on CRF)*	Telehealthand Web/ Mobile-based	9 weeks	Physical Activity, Mindfulness-Based Cognitive Therapy	Effectiveness, Mediators, and Effect Predictors of Internet Interventions for Chronic Cancer-Related Fatigue: The Design and an Analysis Plan of a 3-Armed Randomized Controlled Trial	**Study Protocol** *(Wolvers *et al*. *[23]*, The Netherlands)*	N/A	N/A
**‘Health Navigation’**	Any cancer type *(focus on CRF)*	Web-based	12 weeks	Physical Activity, Sleep, Pain Control, Energy Conservation, Nutrition, Distress Management	Web-Based Tailored Education Program for Disease-Free Cancer Survivors With Cancer-Related Fatigue: A Randomized Controlled Trial	**RCT** *(Yun *et al*. *[35]*, Korea)*	BFI, FSS, HADS, EORTC QLQ-C30	The intervention group demonstrated improvement in fatigue, anxiety, global quality of life and several other aspects of functioning
**‘INSPIRE’** and** ‘INSPIRE + Problem-Solving Treatment (PST)’**	Survivors of Haemato-poietic Stem Cell Trans-plantation	Web-based and Web-based with Telehealth	6 months	‘Boosting Health’ (cardiovascular, bone, second cancer advice), ‘Restoring Energy’ (fatigue, muscle weakness), ‘Lifting Mood’ (depression, distress, social isolation) with or without PST	An online randomized controlled trial, with or without problem-solving treatment, for long-term cancer survivors after hematopoietic cell transplantation	**RCT** *(Syrjala *et al*. *[36]*, USA)*	CTXD, SCL-90-R, SF-36, FSI	For aggregated outcomes, no effects were observed for either intervention. For individual outcomes, INSPIRE + PST was associated with improved distress, INSPIRE alone was marginally associated with improved distress
**‘Lymphoma InterVEntion (LIVE)’**	Lymphoma	Web-based	16 weeks	Psychoeducation, Patient Information (Adverse Physical/ Psychological Problems, Work, Sexuality, Lifestyle)	Lymphoma InterVEntion (LIVE) – patient reported outcome feedback and a web based self-management intervention for patients with lymphoma: study protocol for a randomised controlled trial	**Study Protocol** *(Arts *et al*. *[25]*, The Netherlands)*	N/A	N/A
					Web-Based Return of Individual Patient-Reported Outcome Results Among Patients With Lymphoma: Randomized Controlled Trial	**RCT** *(Oerlemans *et al*. *[37]*, The Netherlands)*	BFI*, EORTC QLQ-C30, HADS, heiQ, MAC, ISQ,	Use of the intervention was low (3%), and an effect could not be determined
**‘Living with Prostate Cancer’**	Prostate Cancer	Web-based and Tele-health	6 months	Physical Activity, Patient Information, Self-Management, Peer Support	Living with prostate cancer: randomised controlled trial of a multimodal supportive care intervention for men with prostate cancer	**Study Protocol** *(Chambers *et al*. *[38]*, Australia)*	N/A	N/A
**‘Oncokompas’**	Head/Neck, Colorectal, Breast, Hodgkin or Non-Hodgkin Lymphoma	Web-based	6 months	Physical Activity, Psychological & Social Functioning, Lifestyle, Existential Issues, Topics In Tumour-Specific Modules	Efficacy, cost-utility and reach of an eHealth self-management application 'Oncokompas' that helps cancer survivors to obtain optimal supportive care: study protocol for a randomised controlled trial	**Study Protocol** *(van der Hout *et al*. *[26]*, The Netherlands)*	N/A	N/A
					Role of eHealth application Oncokompas in supporting self-management of symptoms and health-related quality of life in cancer survivors: a randomised, controlled trial	**RCT** *(van der Hout *et al*. *[39]*, The Netherlands)*	PAM, EORTC QLQ-C30 (SumSC)	After 6 months, Oncokompas was not found to be associated with improvements in levels of patient activation but did increase HRQOL
					The eHealth self-management application ‘Oncokompas’ that supports cancer survivors to improve health-related quality of life and reduce symptoms: which groups benefit most?	**Original Article** *(van der Hout *et al*. *[40]*, The Netherlands)*	EORTC QLQ-C30, FCCHL, GSE, MAC, PAM, PSM,	For symptom reduction, the intervention was most effective for head/neck & colorectal cancer survivors with a higher burden of tumour-specific symptoms. For HRQOL, it was for participants with lower self-efficacy/higher personal control/higher health literacy
					Reasons for not reaching or using web-based self-management applications, and the use and evaluation of Oncokompas among cancer survivors, in the context of a randomised controlled trial	**Original Article** *(van der Hout *et al*. *[41]*, The Netherlands)*	Usage and reasons for using/not using/not participat-ing in the RCT were explored	No symptom burden, no supportive care needs, lack of time were cited as reasons by non-users. Users referenced cancer-generic and tumour-specific materials indicating the value of including a wide range of topics
					Cost-utility of an eHealth application ‘Oncokompas’ that supports cancer survivors in self-management: results of a randomised controlled trial	**Original Article** *(van der Hout *et al*. *[42]*, The Netherlands)*	iMCQ, iPCQ, EQ-5D	The intervention was deemed to be cost-effective; equally effective on utilities, and not more expensive, than care-as-usual
**‘Pack Health’**	Any cancer type *(focus on Pain and CRF)*	Telehealth	8 weeks	Patient Information, Physical Activity, Nutrition, Fatigue, Finance Management, Caregiver Guide	Feasibility of a Telehealth Educational Program on Self-Management of Pain and Fatigue in Adult Cancer Patients	**Feasibility Study** *(Rocque *et al*. *[21]*, USA)*	Feasibility, PAM, MDASI, NCCN (DT), SF-12	Due to the low engagement level (34%), the programme did not meet feasibility criteria
**‘PC Essentials’**	Prostate Cancer	Telehealth	4 weeks + booster session after 3 months	Physical Activity, Patient Information, Psychoeducation	Prostate Cancer Survivorship Essentials for Men with Prostate Cancer on Androgen Deprivation Therapy (ADT)	**Study Protocol** *(Green *[43]*, Australia)*	N/A	N/A
**‘PERC’**	Prostate Cancer *(couples-only)*	Web-based	15 weeks	Relationships & Communication, Symptom Managment (e.g., urinary/ bowel, sexual/hormonal problems, pain, fatigue, sleep disturbance, stress) Improving Healthy Behaviours	Enhancing survivorship care planning for patients with localized prostate cancer using a couple-focused web-based, mHealth program: the results of a pilot feasibility study	**Feasibility Study** *(Song *et al*., *[44]*, USA)*	Feasibility (recruit-ment & retention), FACT-G, EPIC-26, RDGS-21, CSS-9, PROMIS, CCI-B	Intervention group patients reported greater programme satisfaction and better urinary symptom scores than control group patients. Most other primary outcome comparisons were nonsignificant. The intervention was reported as being feasible
**‘PROGRESS’**	Prostate Cancer	Web-based	Missing data	Treatment Type, Physical Side Effects (urinary and sexual dysfunction), Emotional & Interpersonal Concerns (fear of recurrence), Practical Concerns (follow-up care, financial needs), Healthy Lifestyle (nutrition, physical activities)	Development and preliminary testing of PROGRESS: a Web-based education program for prostate cancer survivors transitioning from active treatment	**Original Article** *(Miller *et al*. *[45]*, USA)*	Qualitative Interviews & Usability Testing	Participants expressed interest in content on treatment side effects, body image, emotional/communication difficulties, daily living activities and health skills
**‘REBOOT’**	Survivors of Bone Marrow Transplants	Telehealth	9 weeks	Physical Activity, Nutrition, Behaviour Change Techniques to support Self-Management	Rehabilitation after bone marrow transplant- what is the impact on patient outcomes? The REBOOT trial	**Study Protocol** *(Denehy *et al*. *[46]*, Australia)*	N/A	N/A
**‘ReCHARGE’**	Haematological Cancer (*focus on CRF*)	Web-based	12 weeks	Patient Information, Sleep, Energy Management, Physical Activity, Mind–Body Interventions (CBT and MBT)	ReCHARGE—Online self-management of cancer-related fatigue: a multimodal approach	**Study Protocol** *Avery *et al*. *[47]*, Australia)*	N/A	N/A
**‘RESTORE’**	Any cancer type *(focus on CRF)*	Web-based	6 weeks	Physical Activity, Nutrition, Self-Management, Sleep, Home and Work Life, Thoughts and Feelings, Communication	RESTORE: an exploratory trial of an online intervention to enhance self-efficacy to manage problems associated with cancer-related fatigue following primary cancer treatment: study protocol for a randomized controlled trial	**Study Protocol** *(Grimmett *et al*. *[27]*, UK)*	N/A	N/A
					Managing fatigue after cancer treatment: development of RESTORE, a web-based resource to support self-management	**Original Article** *(Foster *et al*. *[48]*, United Kingdom)*	N/A *(describing the resource develop-ment)*	N/A
					A web-based intervention (RESTORE) to support self-management of cancer-related fatigue following primary cancer treatment: a multi-centre proof of concept randomised controlled trial	**RCT** *(Foster *et al*. *[49]*, UK)*	BFI, CS-SES, FACT-G, PHQ-9, PSEFSM, PWI	The intervention was deemed feasible and acceptable Although there was higher fatigue self-efficacy demonstrated in the intervention versus control group, this difference decreased by 12 weeks
**‘RISE’**	Any cancer type *( focus on CRF)*	Telehealth	Missing data	Energy Conservation, Physical Activity, Cognitive Behavioural Therapy, Sleep, Mindfulness, Nutrition	Cancer-Related Fatigue Protocol Using a Personalized Self-Management Program	**Study Protocol** *(Goodfellow *et al*. *[50]*, USA)*	N/A	N/A
**‘SafeFit’**	Any cancer type	Telehealth& Web-based	6 months	Physical Activity, Nutrition, Psychological Support, Behaviour Change Techniques	SafeFit Trial: virtual clinics to deliver a multimodal intervention to improve psychological and physical well-being in people with cancer. Protocol of a COVID-19 targeted non-randomised phase III trial	**Study Protocol** *(Grimmett *et al*. *[51]*, UK)*	N/A	N/A
**‘SmartManage’**	Sexual Minority Men with HIV and Cancer	Web-based	10 weeks	Relaxation, Physical Activity, Social Support, Patient Information, Psychoeducation, CBT	An Adapted Cognitive Behavioral Stress and Self-management Intervention for Sexual Minority Men Living With HIV and Cancer Using the SmartManage eHealth Platform: Protocol and Study Design	**Study Protocol** *(Puccinelli *et al*. *[52]*, USA)*	N/A	N/A
**‘Simultaneous stage-matched exercise and diet (SSED) intervention’**	Breast Cancer	Telehealth	12 weeks	Physical Activity, Nutrition, Telephone Counselling	Randomized Pilot Test of a Simultaneous Stage-Matched Exercise and Diet Intervention for Breast Cancer Survivors	**Randomised Pilot Study** (Kim [53], Korea)	Feasibility, BFI, EORTC QLQ-C30 (Korean Version), DQI, HADS, IPAQ, Stage of Motiva-tional Readiness	The intervention was found to be feasible and acceptable. Preliminary results indicated that the intervention group had significantly greater improvements in motivational readiness (e.g., physical activity and nutrition), emotional functioning, fatigue and depression
**‘STAMP’**	Any cancer type *(focus on pain)*	mHealth	Missing data	Medication, Constipation Management, Pain Psychology Principles, Health Behaviours and Pain, Skills Training	Leveraging mobile health technology and research methodology to optimize patient education and self-management support for advanced cancer pain	**Original Article** *(Azizoddin *et al*. *[22]*, USA)*	N/A *(describing the resource develop-ment)*	N/A
**‘Surviving and Thriving with Cancer’**	Any cancer type	Web-based	6 weeks	Self-Management, Psychoeducation, Sleep, Fatigue, Physical Activity, Nutrition, Patient Information, Medication, Treatment Side Effects	Surviving and Thriving With Cancer Using a Web-Based Health Behavior Change Intervention: Randomized Controlled Trial	**Original Article** *(O'Carroll Bantum *et al*. *[54]*, USA)*	BFI, BFFQ, GEQ, PHQ-8, WHIIRS	6 months post-intervention, the intervention group had significantly greater reductions in insomnia and increases in physical activity. However, there were no other significant differences observed
**‘TEMPO’**	Prostate Cancer *(couples-only)*	Web-based	10 weeks	Self-Management, Communication, Lifestyle/ Behavioural Change, Psychoeducation, Symptom Management	A study protocol for a multicenter randomized pilot trial of a dyadic, tailored, web-based, psychosocial, and physical activity self-management program (TEMPO) for men with prostate cancer and their caregivers	**Study Protocol** *(Lambert *et al*. *[55]*, Canada)*	N/A	N/A
**Patient Information Leaflet**	Abdominal Cancer *(Colorectal/* *Ovarian)*	Paper-based	1–2 weeks prior to surgery	Physical Activity, Nutrition, Relaxation, Smoking Cessation, Alcohol Cessation	Prehabilitation in cancer care: patients’ ability to prepare for major abdominal surgery	**Original Article** *(Beck *et al*. *[20]*, Denmark)*	Qualitative interviews and adherence records	The authors concluded from the combined adherence and interview data that the leaflet functioned as a tool to motivate and support patients’ prehabilitation-related actions
**WEST**	Breast Cancer	Web-based	24 weeks	Physical Activity, Nutrition, Telephone Counselling	Effects of a web-based expert support self-management program (WEST) for women with breast cancer: A randomized controlled trial	**RCT** *(Kim & Kim *[56]*, Korea)*	HS-SES, Physical measures (e.g., height, weight)	At 24 weeks, the intervention group demonstrated greater decreases in body fat/percentage and waist circumference but no differences were found for perceived self-efficacy
**WSEDI**	Breast Cancer	Web-based	12 weeks	Physical Activity, Nutrition, Self-Management Skills	A Web-based self-management exercise and diet intervention for breast cancer survivors: Pilot randomized controlled trial	**Pilot RCT** *(Lee *et al*. *[57]*, Korea)*	BFI, DQI, EORTC-QLQ-C30, Stage of Motiva-tional Readiness, Perceived Self-Efficacy, Exercise/ Diet	The intervention group was associated with higher levels of exercise, fruit and vegetable intake, HRQOL, fatigue, motivational readiness and aspects of self-efficacy

Abbreviations: BFI* Big Five Inventory, *BFI* Brief Fatigue Inventory, *BFFQ* Block Food Frequency Questionnaire, *BMI* Body Mass Index*, CBT* Cognitive Behavioural Therapy*, CCI-B* 13-item Charlson Comorbidity Index-Brief*, CIS* Checklist Individual Strength, *CRCI* Cancer Related Cognitive Impairment, *CRF* Cancer Related Fatigue, *CSS* 9-item Cancer Self-Efficacy Scale, *CS-SES* Cancer Survivors’ Self-efficacy Scale, *CTXD* Cancer and Treatment Distress, *DMIRSSC* Dutch Measuring Instruments for Research on Smoking and Smoking Cessation, *DSQFC* Dutch Standard Questionnaire on Food Consumption, *DQI* Diet Quality Index, *EORTC QLQ-C30* (SumSC) European Organization for Research and Treatment of Cancer Quality of Life Questionnaire-C30 (Summary Score), *EQ-5D* EuroQol-5 Dimension, *EPIC* Expanded Prostate Cancer Index Composite, *FACTCog* Functional Assessment of Cancer Therapy-Cognition, *FACT-G* 27-item Functional Assessment of Chronic Illness Therapy General Scale, *FCCHL* Functional, Communicative and Critical Health Literacy Scale, *FSI* Fatigue Symptom Inventory, *FSS* Fatigue Severity Scale, *GEQ* Godin Exercise Questionnaire, *GSE* General Self-Efficacy scale, *HADS* Hospital Anxiety and Depression Scale, *heiQ* Health Education Impact Questionnaire, *HRQOL* Health Related Quality of Life, *HS-SES* Health-Specific Self-Efficacy Scale, *iMCQ* Institute for Medical Technology Assessment, *iPCQ* Productivity Costs Questionnaire, *IPQA* International Physical Activity Questionnaire, *ISQ* 9-item Information Satisfaction Questionnaire, *MAC* 40-item Mental Adjustment to Cancer Scale, *MBT* Mindfulness –Based Therapy, *MDASI* MD Anderson Symptom Inventory,, *MHLC* Multidimensional Health Locus of Control Scale, *NCCN* (DT) Modified NCCN Distress Thermometer, *PAM* Patient Activation Measure, *PHQ-8* (-9) Patient Health Questionnaire, *PROMIS* Patient-Reported Outcomes Measurement Information System, *PSEFSM* Perceived Self-Efficacy For Fatigue Self-Management, *PSM* Pearlin and Schooler Mastery, *PWI* Personal Wellbeing Index, *RCT* Randomised Control Trial, *RDGS* 21-item Risk of Distress General Symptom Scale, *SCL-90-R* Symptom Checklist-90-R, *SF-36* Short Form (36) Health Survey, *SF-12* Short Form (12) Health Survey, *SQUASH* Short Questionnaire to Assess Health Enhancing Physical Activity, *UCLA-LS* University of California Los Angeles-Loneliness Scale, *WHIIRS* Women’s Health Initiative Insomnia Rating Scale

Of the six studies that examined feasibility, outcomes were mixed. Three interventions were deemed by authors to be feasible and acceptable [44, 48, 53]. Two studies reported that intervention usage was low, ranging from 3 to 34% of participants assigned to the intervention group [21, 37]. Only one study examined cost-effectiveness: the intervention was found to be equally effective and not more expensive than care-as-usual [26]. These findings are presented in detail in Table 2.

### Adherence to cardio-oncology principles

The included articles were searched individually for references to cardiotoxicity and adherence to the key recommendations from cardio-oncology literature (primarily, that interventions employ evidence-based methods of preventing, detecting, monitoring and treating cardiac risk factors). One intervention [36] reported cardiovascular health as being a subtopic within its syllabus. It was not possible to acquire further information regarding the extent to which cardiovascular health was explored, however, and no references to cardiotoxicity and cardio-oncology guidelines were found within the article. Similarly, the search did not identify any other articles that incorporated the implications of cardiotoxicity or were oriented to cardio-oncology principles.

## Discussion

The aim of this scoping review was to provide a comprehensive synthesis of the current home-based, multimodal, self-management interventions (HMSIs) available for cancer patients. The primary areas of interest included the nature, extent and remit of existing HMSIs, potential obstacles to their implementation and the clarification of key terms and concepts. HMSIs will also be discussed in the context of the current literature on cardio-oncology.

The scoping review included 41 studies, representing 28 HMSIs. There was significant variation amongst HMSIs in terms of delivery mode, duration, location and target. Most HMSIs were ‘web-based’, which referred to the use of an online, independent platform that patients were able to access for a specific amount of time. The literature differentiated between these ‘web-based’ platforms and interventions that employed video conferencing technology: while the latter are also ‘web-based’, due to the live and interactive nature of delivery, the term ‘telehealth conferencing’ was generally applied to differentiate between both mediums. While all ‘web-based’ interventions can be accessed via mobile devices with internet connections, ‘mHealth’ or ‘mobile-based’, interventions designed specifically for a mobile devices were rarer, as were paper-based interventions. Although a growing body of evidence demonstrates the efficacy of digital interventions, the existing literature tends to bind both web- and mobile-based interventions together (e.g., in Kählke et al. [58]). As noted by Lorca-Cabrera et al. [59] future research may benefit from investigating the effectiveness of mobile-based interventions to ascertain which mediums are most accessible and successful for the delivery of home-based interventions, particularly for cancer patients.

There was a similar disparity observed between the duration of interventions. All of the interventions, with the exception of one; The Patient Information Leaflet [20] were within the range of one to six months in length. The Patient Information Leaflet, a one to two-week long intervention, was also the only intervention to focus exclusively on cancer ‘prehabilitation’ – a term defined as “a process on the continuum of care that occurs between the time of cancer diagnosis and the beginning of acute treatment” [60], p. 715]. Given that cancer prehabilitation has been identified as leading to better functional outcomes for cancer patients [61] this finding indicates that perhaps more interventions should focus on providing support earlier in the cancer care pathway and incorporating the prehabilitation period into their timeframe.

One notable finding was that of the 28 interventions included, only two were located within the UK; considerably fewer than the 12 in the US and half the number based in Korea and the Netherlands respectively. The two UK-based interventions identified were ‘RESTORE’ [48] and ‘SafeFit’ [51]. RESTORE was developed by the University of Southampton, in collaboration with Macmillan Cancer Support. An online, multimodal intervention that targets cancer-related fatigue, it is available for free for at https://macmillanrestore.org.uk/. ‘SafeFit’ is a remote trial, designed in response to the COVID-19 pandemic as a ‘virtual clinic’, enabling cancer patients to maintain contact with a cancer exercise specialist, to support their physical and psychological wellbeing. As of August 2023, the SafeFit website states “due to overwhelming demand SafeFit is currently at capacity, and we are unfortunately not able to take any more referrals at this time” (accessed at: https://safefit.nhs.uk/, 22nd August 2023). The scoping review did not identify any interventions targeting all six modalities and generalisable to all cancer types based in the UK.

Only four of 28 interventions included were non-cancer specific and generalisable to any cancer type; ‘Cancer Aftercare Guide’ [24]‘Cancer Thriving and Surviving’ [31] ‘SafeFit’ [51] and ‘Surviving and Thriving with Cancer’ [54]. None of these interventions included all six modalities identified by the thematic analysis (Physical Activity, Nutrition, Psychological Support, Patient Education, Lifestyle and Caregiver Support). In fact, the six modalities were only encompassed by one intervention: ‘Pack Health’ [21], an 8-week telehealth programme targeting cancer-related fatigue, which due to low engagement level, did not meet feasibility criteria. While all interventions included a psychological component (as necessitated by the inclusion criteria), the majority included at least three other components but these varied significantly between Physical Activity, Lifestyle, Nutrition and Patient Education. Only two interventions included a Caregiver Support component. A previous systematic review found that although the research on multidimensional cancer interventions is scarce, evidence has shown “statistically significant benefits for multidimensional interventions over usual care, most notably for the outcomes fatigue and physical functioning” [62]. However, it is clear from this scoping review that the term ‘multidimensional’ is ambiguous. If the term is to be defined using the ‘multidimensional’ interventions of cardiac rehabilitation, all six components identified should be incorporated, including a Caregiver Support section (e.g., the ‘Friends and Family Resource’ of the REACH-HF Heart Failure Manual programme [63, 64]).

Breast, prostate and haematological cancers were the most common types of cancer targeted by the interventions included in this scoping review. This finding can likely be traced back to an analysis of the distribution of cancer research spending from 2012, which showed that breast cancer, prostate cancer and leukaemia were funded at levels that appeared higher than their relative burden, while other cancers (e.g., bladder, oesophageal, liver and uterine cancers) were underfunded [65]. Fatigue has been reported as the most common symptom experienced by cancer patients, which, in conjunction with its persistent, distressing and debilitating nature [66] likely explains its prevalence as a subject of interventions. This finding would also indicate that even those interventions not solely focused on cancer-related fatigue, would benefit by incorporating it as a target component. Only four interventions were generalisable to all cancer patients. An interesting observation is that despite the wide distribution of cancer types and related symptoms targeted by the interventions, each consisted of an assortment of same six aforementioned modalities, indicating that regardless of cancer type, the same information (in terms of psychological support, physical activity, lifestyle, etc.) is relevant. In order to address the skewed distribution of cancer interventions, perhaps the future of multidimensional cancer survivorship care lies in the development of comprehensive, generalisable interventions that can be tailored, where necessary, to meet specific patient needs.

Surprisingly, no intervention included in the present review appeared to be oriented to the literature on cardiotoxicity and cardio-oncology guidelines. One intervention ‘INSPIRE’ [36] a programme for cancer survivors of hematopoietic cell transplantation, mentioned cardiovascular health as being a topic within its ‘Boosting Health’ module, but failed to reference cardiotoxicity or cardio-oncology guidelines. Similarly, from the information available, the remaining interventions did not appear to target the key cardio-oncology recommendations of preventing, detecting, monitoring and treating cardiac risk factors, with regard to the implications of cardiotoxicity in particular. These findings are concerning, given that the research documenting elevated levels of cardiovascular risk factors in cancer survivors has been available for over a decade [67–69]. In 2013, Weaver et al. reported that of cancer survivors, “62.0% were overweight or obese; 55.0% reported hypertension; 20.7% reported diabetes; 18.1% were inactive; and 5.1% were current smokers” [69], p. 1]. Certain risk factors such as obesity, hypertension and age are baseline in nature. However, as previously outlined, others occur as a result of injury from cancer treatment: both directly (e.g., cardiotoxicity) and indirectly (e.g., deconditioning and weight gain) [11]. This raises important questions regarding the ethical viability of delivering treatments with potentially critical side-effects, without providing the necessary care to mitigate these risk factors.

Indeed, literature has long been highlighting the need for comprehensive and integrative survivorship care for cancer patients with cardiovascular comorbidities. As noted by Sparano & Sahni [70] in relation to the recent ESC Guidelines [8] “there is no shortage of similar guidelines dating back to as early as 2014”. The guidelines have not only included detailed, evidence-based recommendations for cardio-oncological support but also described pathways for facilitating these recommendations based on existing models of care. Early articles that defined the emergent field of cardio-oncology emphasised that a collaborative approach between cardiologists and oncologists would be required [67–69, 71]. Subsequent papers identified that cardiac rehabilitation frameworks may provide a useful structure to underpin future cardio-oncology interventions. Most recently, a statement from the American Heart Association outlined explicit cancer-specific adaptations for each factor targeted by cardiac rehabilitation, from patient assessment, nutrition counselling, tobacco cessation, exercise training, to weight, diabetes mellitus, lipid, blood pressure and psychosocial management [11]. Since then, the “underrated relationship” between oncology and cardiac rehabilitation [72]. p. 1] has increasingly been acknowledged. However, the results of the present scoping review would suggest that despite the significant volume of literature, the knowledge we have about supporting cancer survivors has yet to be successfully translated from theory to practice. In order to successfully bridge this gap in survivorship care, future research and interventions must be informed by cardio-oncology principles and guidelines, and focus on adapting well-established, evidence-based models of cardiac rehabilitation.

The PRISMA-ScR Guidelines [19] were implemented in order ensure the present study was conducted as accurately and effectively as possible. Nonetheless, this scoping review has some limitations. In order to make the literature search more feasible, a relatively specific search term was developed and, given the sizeable number of results, the bibliographies of included studies were not searched for further studies. This was justified by the research team as being in concordance with the Levac et al., framework for scoping reviews which emphasises balancing “feasibility with breadth and comprehensiveness” [73], p. 1]. Similarly, a critical appraisal of the included sources of evidence was also not conducted, as this was not deemed relevant to the objectives of this scoping review.

## Conclusion

While a considerable number of study protocols and pilot studies exist documenting HSMIs, fewer appear to have progressed beyond this stage of development. Of those that have, the present review did not identify any ‘feasible’ interventions that covered each of the six modalities included in models of multidimensional cardiac care, while being generalisable to all cancer survivors and incorporating the recommendations from cardio-oncology research and evidence. Recent publications have been raising awareness of the “underutilization of preventive cardiovascular measures in patients with cancer” [74], p. 1325] and this “unrecognized opportunity to improve survival in cancer patients” [75], p. 1323]. Given increasing risks of cardiotoxicity and resulting fatality in this already vulnerable population, and the longstanding efficacy of cardiac rehabilitation programmes in tackling the very risk factors identified by cardio-oncology literature, there is certainly an opportunity, if not necessity, for cardiac rehabilitation to expand to meet the needs of those living with and beyond cancer.

## Data Availability

All data generated or analysed during this study are included in this article.
